# Supplementary material for: Sketching methods with small window guarantee using minimum decycling sets
Source: ArXiv. 2023 Nov 6:arXiv:2311.03592v1. Preprint. [Version 1] (PMC10659450)
Supplement: 1 [file NIHPP2311.03592V1-supplement-1.pdf]

Supplementary Material

1 Exponential window guarantee for Decycling Sets (DS)

Consider a syncmer sketching method selecting a k-mer if the smallest s-mer is at position 1 (first position). Assume $s \leq k-1$. The order on the s-mer is as follows: create a de Bruijn sequence D of order s (it contains all the s-mers once and only once) and $s_1 < s_2$ iff the s-mer s_1 appears after s_2 in D. The sequence D is a decreasing sequence of s-mers of length $\sigma^s + s - 1$. With $s = k-1$, we created a sequence of length $\Omega(\sigma^{k-1})$ without a selected k-mer.

2 MDS graph structure

Lemma 1 (Commutative property) *Let M be an MDS and $f_1, f_2 \in \Sigma^{k-1}$ be two valid F-moves in M, then f_1 is a valid F-move in f_2M, f_2 is valid in f_1M, and $f_1f_2M = f_2f_1M$.*

Proof. The left companions of f_1 and f_2 are all in different PCRs. Hence, after doing the F-move f_1 or f_2, the other F-move is still valid. Moreover, regardless of the order in which the F-moves are performed, the resulting set is the same. ■

By extension, in a chain of F-moves, reordering the F-moves, as long as it is valid, does not change the result. Note that there is no equivalent statement for I-moves: if $f_1|_{m_1}, f_2|_{m_2}$ are two valid I-moves in M, then $f_2|_{m_2}$ may not be valid in $f_1|_{m_1} M$.

In the following proofs, we use the simplified representation for PCRs, F- and I-moves given in Figure 4. For simplicity, the figure shows an example with the binary alphabet. When $\sigma > 2$, an F-move f represents a hyperedge between σ PCRs rather than a simple edge as shown.

Proposition 3 ($G_{\mathbf{MDS}}$ component structure) *For any σ and k, the components of $G_{MDS}(\sigma, k)$ satisfy:*

1. *every component is strongly connected*
2. *every cycle is of length $\alpha\sigma^{k-1}, \alpha \in \mathbb{N}$*
3. *in a cycle of length $\alpha\sigma^{k-1}$, every possible F-move $f \in \Sigma^{k-1}$ occurs exactly α times*
4. *every node is in a cycle of length σ^{k-1} (hence the girth is σ^{k-1})*
5. *each component is a σ^{k-1}-partite directed graph*

Proof *Points 2 and 3, length of cycles.* Every PCR is a cycle in D_k and an MDS M is seen as pebbles sitting on the k-mers (see Figure 4 b) There is one pebble per PCR. An F-moves involves σ distinct PCRs (edges $af \to fa, a \in \Sigma$ are each in their own PCR). Hence an F-moves is an hyperedge connecting σ PCRs. An F-move is like moving the pebbles along σ PCRs at a time, from left-companions to right-companions, and this move is legal only if $\mathrm{lc}(f) \subset M$. In that sense, an F-move is like a semaphore: pebbles can move only if all their left-companions are present in the set.

First, because every MDS has a valid F-move and a component of G_{MDS} is finite, a component must have a cycle. Let $C = (M_0, \ldots, M_{n-1})$ be a cycle of MDSs in G_{MDS}, and equivalently

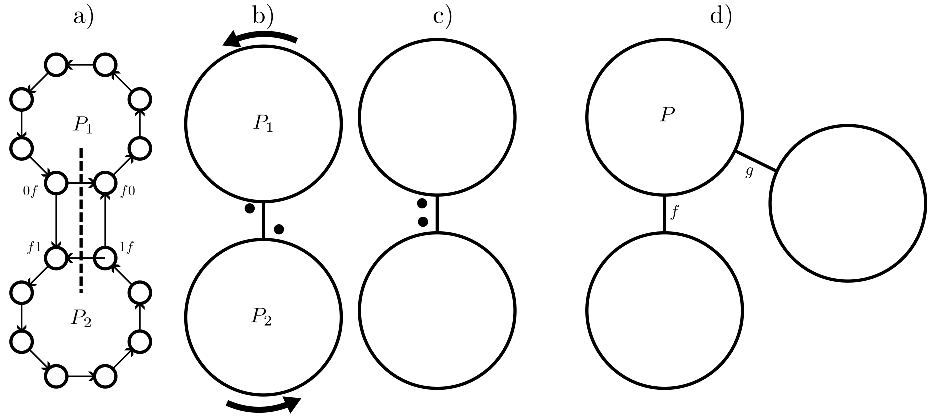

Figure 4: Simplified representation of PCRs, F-moves and I-moves when $\sigma = 2$. a) shows two PCRs from the de Bruijn graph D_k. Every k-mer is a circle, and they are all oriented counter-clock-wise (see PCR P_1 and P_2 here). Let f be an F-move that involves P_1, P_2. Here P_1 has the edge $0f \to f0$, and P_2 has $1f \to f1$: these are the PCR edges. The cross-PCR edges $0f \to f1$ and $1f \to f0$ form anti-parallel edges between P_1 and P_2. b) The *simplified PCR/pebbles* representation shows PCRs as large cycles without representing individual k-mers and only representing the F-move edges of interest. The elements from the MDS in each PCR (the pebbles) are small black circles that can travel only counter-clock-wise around the PCR. An F-move is an edge between P_1 and P_2 and act as a semaphore: a pebble can move one step around the PCR and across the edge of f only when the other pebbles are present next to the edge in the other PCR (i.e., $\mathrm{lc}(f)$ is in the MDS), as shown in b), and all pebbles move across the edge at the same time. c) The position of the pebbles for the I-move $f|_1$: bit 0 is set but not bit 1, so the pebbles are on $0f$ and $f1$ (left side of the edge of f). The top pebble can move across the edge, counter-clock-wise, while the lower one stays still. For I-move $f|_2$ with bit 0 unset and bit 1 set, the pebbles would be on $1f$ and $f0$, on the right side of the edge of f. d) If F-moves f and g have a PCR P in common, then, because F-moves act like semaphores, it is not possible to do the F-move f twice before g is done once. For the pebble to go around P to do f a second time, necessarily the F-move g was done as well.

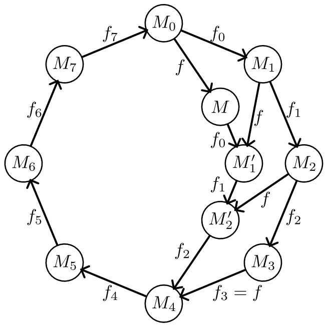

Figure 5: Example of a cycle in $G_{\mathrm{MDS}}(2,4)$. The outer circle is $C = (f_0, \ldots, f_7)$, a cycle of length σ^{k-1}. $M = fM_0$ is a neighbor of M_0 not on C. Because f must occur in C, here $f = f_3$, then f commutes with f_0, f_1, f_2. Hence $(f = f_3, f_0, f_1, f_2, f_4, \ldots, f_7)$ is also a cycle in $G_{\mathrm{MDS}}(2,4)$ and it contains M_0 and M.

$C = (f_0, \ldots, f_{n-1})$ is a list of F-moves such that $M_{i+1} = f_i M_i$ (indices taken modulo n). After doing F-move f_0, the pebble on at least one PCR, say P_0, has moved. Because C is a cycle, by the time f_{n-1} is done, all pebbles are back on their respective starting spot. Meaning the pebble on P_0 went all the way around (possibly multiple times) P_0. To move around P_0 with F-moves, the pebbles in the PCR adjacent to P_0 must have moved as well, and, by the time f_{n-1} is done, go around their respective PCRs. By transitivity, and because the de Bruijn graph is strongly connected, every pebble on every PCR has gone around its PCR after f_{n-1} is done. Because every node went around its PCR, this means that every one of the σ^{k-1} F-moves was done and $n \geq \sigma^{k-1}$.

Conversely, because the F-move/hyperedge act as semaphores, it is not possible for a pebble on a PCR to do more rotations around its own PCR than the pebbles on the adjacent (by hyperedge) PCRs. To see this, consider the starting position of the pebble on PCR P_0. For this pebble to start a second turn around P_0, all of its left-companions must be back on their starting spot and also start a second turn around their own PCRs. This holds for all PCRs by transitivity.

Hence, in a cycle of the MDS graph, the pebbles of all PCRs go around the same number of times, say α, and the number of F-moves in the cycle C is $n = \alpha\sigma^{k-1}$. ■

Proof *Point 1, strongly connected.* As in the previous proof, there exists a cycle $C = (M_0, \ldots, M_{n-1})$ in G_{MDS}, and its edges are $(f_0, \ldots, f_{n-1})$ with $M_{i+1} = f_i M_i$.

We show that for any node M_i of this cycle and any neighbor M of M_i, reachable by an F-move or RF-move from M_i, M and M_i are in a cycle. If this holds, by transitivity of the relation "being in the same strongly-connected component", any pair of nodes in the component are in a cycle and the component is strongly-connected.

WLOG, let's prove it for M_0 (see Figure 5). It is a consequence of the commutativity of the F-moves (Lemma 1). Let $M = fM_0$ be a neighbor of M_0 for some $f \neq f_0$. Because in a cycle all F-moves occur, there exists a first $j \in [1, n-1]$ such that $f_j = f$ (and $f \neq f_i, i \in [0, j-1]$). f is valid in M_0, hence it is also valid in M_1, and recursively in , $M_2, \ldots, M_j$. Therefore f commutes with $f_0, \ldots, f_{j-1}$ and the series of F-move $(f = f_j, f_0, \ldots, f_{j-1})$ is another path from M_0 to M_{j+1} that is going through M. This path followed by the remainder of C from M_{j+1} back to M_0 is a cycle that includes both M_0 and M. ■

Proof *Point 4, cycle length σ^{k-1}.* Let M be an MDS on a cycle C in G_{MDS}. It is of length $\alpha \cdot \sigma^{k-1}$, with $\alpha \geq 1$ by point 2. Suppose that $\alpha > 1$. Let $C = (f_1, \ldots, f_{\alpha \cdot \sigma^{k-1}})$ be the chain of

F-moves representing that cycle. Every distinct F-move occurs exactly α times in that chain. We show that the chain can be reordered so that the σ^{k-1} different F-moves occur at the first σ^{k-1} positions of the chain.

If it is not already the case that the first σ^{k-1} F-moves are distinct, there must be an F-move f that occurs twice in the list before an F-move g occurs for the first time. Let $i < j$ be two indices which are the first two occurrences of f in the chain (i.e., $f_i = f_j = f$), and such that $j+1$ is the first occurrence of g ($f_{j+1} = g$). If any of the PCRs involved in the F-move f are also involved in the F-move g, then it is not possible to use f twice in C before using g (see Figure 4d). Therefore the PCRs involved in the F-moves f and g are distinct, and g must be a valid F-move just before the second use of f as well. In other words, f_j and f_{j+1} commute.

Repeated swapping of F-moves leads to the desired chain of F-moves with all σ^{k-1} distinct F-moves in the first positions, which induces a cycle of length σ^{k-1} containing M. ∎

Proof *Point 5, σ^{k-1}-partite.* Partition the nodes of a component of G_{MDS} as follows. We create σ^{k-1} sets: $\mathcal{P}_0, \ldots, \mathcal{P}_{\sigma^{k-1}-1}$. Let M_0 be an arbitrary MDS of the component and assign it to the set $\mathcal{P}_0$. For every other MDS M, take a shortest path $P(M) = M_0 \to M$ in G_{MDS}. Assign M to the partition with index $|P| \mod \sigma^{k-1}$.

Because M_0 is in a cycle of length σ^{k-1}, every set $\mathcal{P}_i$ has at least one MDS assigned to it. Moreover, every MDS is assigned to exactly one set. Hence the sets $\mathcal{P}_i$ form a partition of the MDSs in the component.

An edge between MDSs in sets $\mathcal{P}_i$ and $\mathcal{P}_j$ with $j > i+1$ would imply the existence of a cycle containing M_0 of length $< \sigma^{k-1}$, which is not possible. ∎

3 Cycle signature unique per component

An MDS M is called *f-terminal* if the only valid F-move in M is f.

Lemma 2 *For any $f \in \Sigma^{k-1}$ and in any component of G_{MDS}, there exists an f-terminal MDS.*

Proof. From Proposition 3, in any component there exists an MDS M' where f is a valid F-move. If there exists other valid F-moves than f in M', do them recursively. I.e., we do every possible F-move in M' but refuse to do f. This creates a path P of MDSs in G_{MDS} starting at M' that does not contain f as an edge.

Because every cycle in G_{MDS} contains every possible F-move, P cannot induce a cycle, and it must terminate at an MDS M. By construction M is f-terminal. ∎

An f-terminal MDS M has a useful property: every maximal path in D_k that avoids M (as created by a walk like in Proposition 1) must start at a k-mer $m \in \text{rc}(f)$. Equivalently, any walk in D_k that avoids M following edges backward ends at some $m \in \text{rc}(f)$.

Proposition 4
1. *Let M be an MDS and f a valid F-move in M, then for any cycle C, $\mathcal{H}_M(C) = \mathcal{H}_{fM}(C)$*
2. *For every valid I-move $f|_m$ in MDS M, there exists a cycle C of D_k such that $\mathcal{H}_M(C) \neq \mathcal{H}_{f|_m M}(C)$*
3. *For any MDSes M_1, M_2 from the same component of G_{MDS}, $\mathcal{S}(M_1) = \mathcal{S}(M_2)$*
4. *For any MDSes M_1, M_2 from different components of G_{MDS}, $\mathcal{S}(M_1) \neq \mathcal{S}(M_2)$*

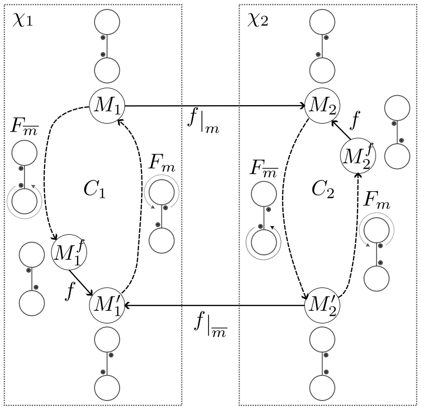

Figure 6: Simplified example for finding the complementary I-moves, when $\sigma = 2$. On the left box, component χ_1 and component χ_2 on the right, of G_{MDS}. The cycle C_1, C_2 are cycles in χ_1 and χ_2 respectively. The simplified PCR/pebble drawings represent the position of the pebbles on the PCRs of P_m (top PCR) and $P_{\overline{m}}$ (bottom PCR). The edge between these PCRs represents f. The PCR/pebbles drawings next to the MDS nodes represent the state of the PCRs for these MDSs, while the drawings next to the F-move lists represent the action of the list of F-moves on the pebbles. From the cycle C_1 in χ_1, we construct cycle C_2 in χ_2 by swapping the order of the F-moves: $(F_{\overline{m}}, f, F_m) \to (F_m, f, F_{\overline{m}})$. These cycles go through the desired MDSs M_2' and M_1' that are linked by the complementary I-move $f|_{\overline{m}}$.

Proof *Point 4, different signatures.* Fix $f \in \Sigma^{k-1}$ and by Lemma 2 we can assume that M_1 and M_2 are both f-terminal, each in its own component. We will construct a cycle C in D_k that has different hitting numbers between the components: $\mathcal{H}_{M_1}(C) \neq \mathcal{H}_{M_2}(C)$.

M_1 and M_2 are in different components, so they are distinct MDSs and there exists a PCR R where the selected k-mer is different. That is, $R \cap M_1 \triangleq m_1 \neq m_2 \triangleq R \cap M_2$. Take a path P in D_k following edges backward from node $0f$ (which is in both M_1 and M_2) to m_1 that avoids nodes $af, a \in \Sigma \setminus \{0\}$. Path P exists because D_k is $(\sigma - 1)$-connected. Because $m_1 \in M_1 \,\Delta\, M_2$, there must exist a first node $m \in P$ which is in $M_1 \,\Delta\, M_2$.

Let P_1 be the restriction of the path P from $0f$ to m and, WLOG, assume that $m \in M_1$. By construction, $|P_1 \cap M_1| = |P_1 \cap M_2| + 1$.

Let P_2 be a path created by a maximal random walk in D_k, following edges backward, starting from m and that avoids M_2. Because M_2 is f-terminal, the walk ends at a node $fa \in \text{rc}(f), a \in \Sigma$. By construction, $|P_2 \cap M_1| \geq |P_2 \cap M_2| = 0$ (P_2 avoids nodes from M_2 but may contain nodes from M_1).

Two cases can happen. First case, there exists a first node $m' \in P_1 \cap P_2$. Then define the cycle C as the restriction of P_1 from m' to m followed by the restriction of P_2 from m to m'. Second case, $P_1 \cap P_2 = \emptyset$ and define the cycle C as the concatenation of P_1, P_2 and backward edge $fa \to 0f$.

In both cases, C satisfies by construction $\mathcal{H}_{M_1}(C) > \mathcal{H}_{M_2}(C)$. ∎

4 G_{comp} is undirected

Proposition 5 (G_{comp} is undirected) *Let $f|_m$ be a valid I-move from MDS M_1 in component χ_1 to M_2 in χ_2. Then there exists M_2', M_1' in χ_2, χ_1, respectively, such that $f|_{\overline{m}}$ (where $\overline{m}$ is the bit-complement of m) is a valid I-move from M_2' to M_1'.*

Proof. See Figure 6. In component χ_1, by Proposition 3, there is a cycle C_1 of length σ^{k-1} that contains MDS M_1, and this cycle has f has an F-move. Hence, $C_1 = (M_1, \dots, M_1^f, M_1', \dots)$ where M_1^f is the MDS where f is a valid I-move and $M_1' = fM_1^f$. Equivalently, looking at the edges, $C_1 = (F_{\overline{m}}, f, F_m)$ where $F_{\overline{m}}, F_m$ are lists of F-moves.

In M_1, $f|_m$ is a valid I-move, which means that if $m_a = 1$, then $af \in M_1$ and $fa \in M_1$ otherwise.

Let's call P_m the set of PCRs that contain af when $m_a = 1$, and $P_{\overline{m}}$ the PCRs containing af when $m_a = 0$ (P_m contains only the top PCR in Figure 6, and $P_{\overline{m}}$ the bottom PCR).

In M_1^f, f is a valid F-move, which means that $af \in M_1^f$ for all $a \in \Sigma$. In other words, the list of F-moves $F_{\overline{m}}$ made by the pebbles in the PCRs in $P_{\overline{m}}$ go around from fa to af, while the pebbles in the PCRs in P_m did not move. (The only way for the pebbles in the PCRs in P_m to move is to do F-move f, which by construction is not in $F_{\overline{m}}$).

Similarly, the list of F-moves F_m made by the pebbles in the PCRs in P_m go around from fa to af, while the pebbles in the PCRs of $P_{\overline{m}}$ did not move.

Now from M_1 do the valid I-move $f|_m$. This advances the pebbles in the PCRs of P_m from af to fa (forward by 1 edge), to get to M_2 in component χ_2, where $\mathrm{rc}(f) \subset M_2$. The position of the pebbles in M_1 and M_2 agree everywhere except on the PCRs of P_m. Because the F-moves in $F_{\overline{m}}$ do not affect the PCRs of P_m, the list $F_{\overline{m}}$ is a valid list of F-moves in M_2 as well.

$fa \in M_2$ for all $a \in \Sigma$. Applying $F_{\overline{m}}$ to M_2 leads to MDS M_2' where $af \in M_2'$ if $m_a = 0$ and $fa \in M_2'$ otherwise. In other words, I-move $f|_{\overline{m}}$ is valid in M_2'. It is easy to check that doing the I-move $f|_{\overline{m}}$ gets back to M_1'.

For completion, one can check that the list of F-moves F_m applies to M_2' because M_2' and M_1' only differs on the pebbles on the PCRs of $P_{\overline{m}}$ and F_m does not affect those PCRs. Applying F_m get to M_2^f where f is a valid F-move and $M_2 = fM_2^f$.

Therefore, the cycle $C_1 = (F_{\overline{m}}, f, F_m)$ is a valid cycle in χ_1 and contains M_1 and M_1', while $C_2 = (F_m, f, F_{\overline{m}})$ is valid in χ_2 and contains M_2 and M_2'. ■

5 Non-decycling PCR sets

Proposition 8 *Let G_{PCR} be the graph with non-decycling PCR sets as nodes and F-moves as edges. Then each component of G is a DAG.*

Proof. Suppose there exists a cycle $\mathcal{C} = \{M_1, \dots, M_n\}$ in G_{PCR}, where $M_{i+1} = f_i M_i$. Because M_1 is not decycling, then there exists a cycle C in $D_k \setminus M_1$. Because RF-moves preserve the hitting number, C is also a cycle in $D_k \setminus f_1 M_1$, and by induction a cycle in $D_k \setminus M_i, i \in [1, n]$. From the proof Proposition 3, any cycle $\mathcal{C}$ must do every σ^{k-1} F-move to return to the starting set, and the union of all the left-companions of the F-moves is the set of all k-mers. This is a contradiction.

6 I-move and constrained cycles

Proposition 6 *Let $f \in \Sigma^{k-1}, m \in [1, 2^\sigma - 2]$, and let χ be a component of G_{MDS}. Then $f|_m$ is not a valid I-move in any MDS of χ if and only if $\exists a, b$ such that $m_a = 1, m_b = 0$ and there exist a constrained cycle using the edge $af \to fb$.*

Proof. Let $f|_m$ be a potential I-move with $m_a = 1$ and $m_b = 0$ ($a, b \in \Sigma, a \neq b$).

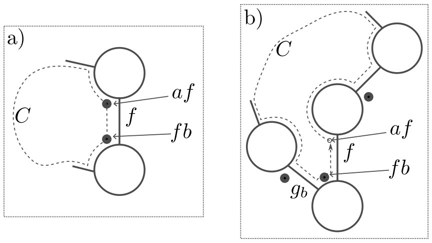

Figure 7: a) The $f|_m$ with $m_a = 1$ and $m_b = 0$ is not possible because $\mathcal{H}_M(C) = 1$. When the I-move $f|_m$ is valid, necessarily C's hitting number must be at least 2. b) Suppose $f|_m$ is never valid, then a backward walk creates a cycle with hitting number 1 using the edge $af \to fb$.

Suppose there exists a constrained cycle C in the de Bruijn graph D_k using the edge $af \to fb$, and $H_\chi(C) = 1$. If $f|_m$ is a valid I-move in an MDS $M \in \chi$, then by definition $af, fb \in M$, hence $H_M(C) \geq 2$. This contradict that C is constrained (see Figure 7 a).

Conversely, suppose that $f|_m$ is not a valid I-move in any MDS of χ. Let $M^f \in \chi$ be an MDS where f is a valid F-move and $M = fM^f$. Then $\mathrm{rc}(f) \subset M$. Define $g_c \triangleq f[2:k-2]c, c \in \Sigma$, that is for all right-companion of f, $fc \in \mathrm{lc}(g_c)$.

From M recursively do all valid F-moves except for the F-moves g_c where $m_c = 0$ to obtain $M' \in \chi$ where the only valid F-moves are exactly those than we refused to do. There must exist $a \in \Sigma$ such that $m_a = 1$ and $af \notin M'$, otherwise $f|_m$ is a valid I-move in M' (see Figure 7b). From af do a walk that avoids M' using backward edges. This walk must end at one of the right-companions of the valid F-moves in M', that is there exists b such that walk ends at $m' \in \mathrm{rc}(g_b)$. By construction there is a backward edge $m' \to fb$. Then follow the backward edge $fb \to af$ to create a cycle C. By construction the only node from M' in cycle C is fb, hence $\mathcal{H}_{M'}(C) = 1$ and C uses the edge $af \to fb$ with $m_a = 1$ and $m_b = 0$. ■
